# Define SNP thresholds for delineation of tuberculosis transmissions using whole-genome sequencing

**DOI:** 10.1128/spectrum.00418-24

**Published:** 2024-06-25

**Authors:** Yu-Xin Xiao, Tai-Hua Chan, Kuang-Hung Liu, Ruwen Jou

**Affiliations:** 1 Tuberculosis Research Center, Centers for Disease Control, Ministry of Health and Welfare, Taipei, Taiwan; 2 Reference Laboratory of Mycobacteriology, Centers for Disease Control, Ministry of Health and Welfare, Taipei, Taiwan; Foundation for Innovative New Diagnostics, Geneva, Switzerland

**Keywords:** tuberculosis, *Mycobacterium tuberculosis*, whole-genome sequencing, transmission, outbreak investigations

## Abstract

For facilitating tuberculosis (TB) control, we used a whole-genome sequencing (WGS)-based approach to delineate transmission networks in a country with an intermediate burden of TB. A cluster was defined as *Mycobacterium tuberculosis* isolates with identical genotypes, and an outbreak was defined as clustered cases with epidemiological links (epi-links). To refine a cluster predefined using space oligonucleotide typing and mycobacterial interspersed repetitive unit variable tandem repeat typing, we analyzed one pansusceptible TB (C1) and three multidrug-resistant (MDR)-TB (C2-C4) clusters from different scenarios. Pansusceptible TB cluster (C1) consisting of 28 cases had ≤5 single nucleotide polymorphisms (SNPs) difference between their isolates. C1 was a definite outbreak, with cases attending the same junior high school in 2012. Three MDR-TB clusters (C2-C4) with distinct genotypes were identified, each consisting of 12–22 cases. Some of the cases had either ≤5 or ≤15 SNPs difference with clear or probable epi-links. Of note, even though WGS could effectively assist TB contact tracing, we still observed missing epi-links in some cases within the same cluster. Our results showed that thresholds of ≤5 and ≤15 SNPs difference between isolates were used to categorize definite and probable TB transmission, respectively. Furthermore, a higher SNP threshold might be required to define an MDR-TB outbreak. WGS still needs to be combined with classical epidemiological methods for improving outbreak investigations. Importantly, different SNP thresholds have to be applied to define outbreaks.

**IMPORTANCE:**

TB is a chronic disease. Depending on host factors and TB burden, clusters of cases may continue to increase for several years. Conventional genotyping methods overestimate TB transmission, hampering precise detection of outbreaks and comprehensive surveillance. WGS can be used to obtain SNP information of *M. tuberculosis* to improve discriminative limitations of conventional methods and to strengthen delineation of transmission networks. It is important to define the country-specific SNP thresholds for investigation of transmission. This study demonstrated the use of thresholds of ≤5 and ≤15 SNPs difference between isolates to categorize definite and probable transmission, respectively. Different SNP thresholds should be applied while a higher cutoff was required to define an MDR-TB outbreak. The utilization of SNP thresholds proves to be crucial for guiding public health interventions, eliminating the need for unnecessary public health actions, and potentially uncovering undisclosed TB transmissions.

## INTRODUCTION

Tuberculosis (TB) is an airborne-transmitted disease caused by *Mycobacterium tuberculosis*, which is one of the leading causes of death worldwide (1). In Taiwan, TB has the highest incidence rate among all human communicable diseases, with an incidence of 28 per 100,000 population in 2022 (2). Controlling TB transmission is key to achieving the goal of ending the TB epidemic by 2030, as proposed by the World Health Organization (WHO) End TB Strategy (3).

Targeted interventions to prevent transmission require the combination of effective genotyping of *M. tuberculosis* strains with enhanced epidemiological investigations. The TB genotyping program has been established for the investigation of presumable outbreaks since 2003 in Taiwan. Initially, conventional genotyping methods, restriction fragment length polymorphism (RFLP) (4), space oligonucleotide typing (spoligotyping) (5), and 15-loci mycobacterial interspersed repetitive unit variable tandem repeat typing (MIRU-VNTR) (6), were applied to define clusters and together with information obtained using structured questionnaires to confirm true outbreaks. To shorten the turnaround time, an optimized 10-loci MIRU was set up to substitute RFLP and 15-loci MIRU in 2015 (7). Nevertheless, the aforementioned methods have limited discriminatory power and may not be able to distinguish genetically closely related strains (8). Whereas whole-genome sequencing (WGS) allows identification of putative index cases, super-spreaders, and routes of transmission (8). To determine genetic relatedness, we studied the optimal number of single nucleotide polymorphisms (SNPs) between sequences of strains. A pre-defined threshold number of SNPs could be applied to include cases in the same putative transmission cluster. Although studies used thresholds ranging from 0 to 50 SNPs, 5 SNPs and 12 SNPs were two widely used thresholds for defining a cluster or recent transmission (9). Nevertheless, appropriate SNP thresholds may be impacted by factors such as local TB incidence, strain type (lineage or subspecies), within-host diversity, duration of infection, and acquisition of drug resistance (9). Currently, there is no international standard for the SNP thresholds to rule in TB transmission (10).

In this study, we investigated the feasibility of a WGS-based approach with defined SNP thresholds and thorough epidemiological investigations to delineate transmission for developing actionable end TB strategies.

## MATERIALS AND METHODS

### Study design

**Fig 1:**
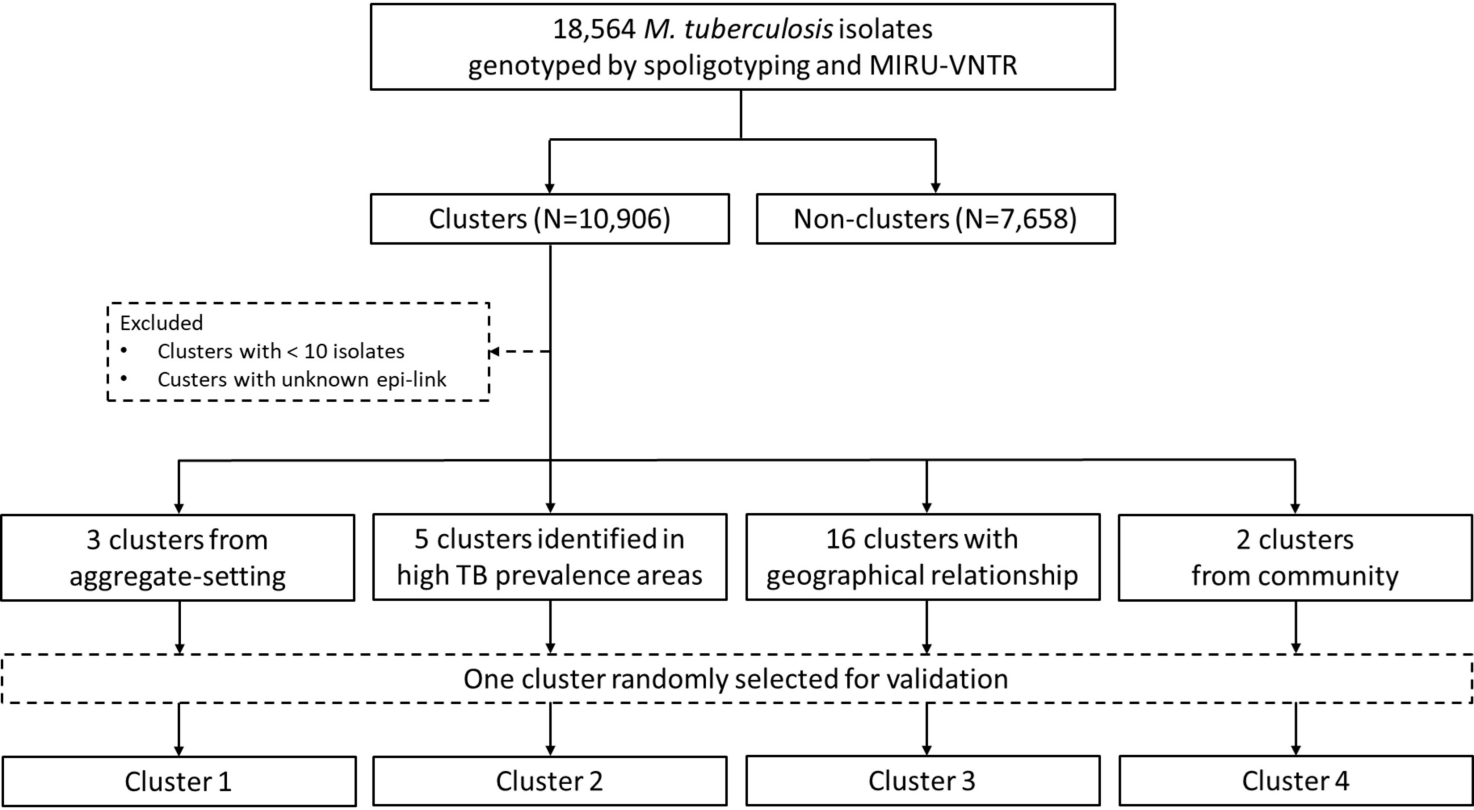
Flowchart of study clusters selection process.

In this study, we selected representative clusters, defined as at least two isolates with identical genotypes, previously defined using spoligotyping and MIRU-VNTR in our genotyping database of 18,564 *M*. *tuberculosis* isolates. Of which, 10,906 isolates were in 2,200 clusters with size ranged from 2 to 268 isolates. Clusters consisting less than 10 isolates or with unknown epi-links were excluded from further analysis. The remaining clusters were categorized into four types, clusters from aggregate-setting (*N* = 3), clusters identified in high TB prevalence areas (*N* = 5), clusters with geographical relationships (*N* = 16), and clusters from community (*N* = 2). One cluster was randomly selected from each of these four types for further validation (Fig. 1). One non-drug-resistant (DR)-TB cluster (Cluster1, C1) from aggregated settings was identified during 2012–2018. Besides, since the majority of multidrug-resistant (MDR)-TB are new cases, we investigated three MDR-TB clusters (Clusters 2–4, C2-4) from high-risk regions or populations consisting of high accumulated cases, different strain genotypes (Beijing, Haarlem, and unknown), contact histories (family, school, workplace leisure, etc.), and geographic relevance. Information on the study cases was obtained from the National TB Registry.

A “confirmed epidemiological link” between two cases was defined as cases knowing each other, or cases sharing time in the same setting during the period when one of the cases was potentially infectious. A “probable epidemiological link” was defined as cases that did not know each other but shared the same geographical areas where transmission probably occurred.

### Spacer oligonucleotide typing (spoligotyping)

Spoligotyping was used for genotyping. A commercially available kit (Ocimum Biosolutions, India) was used as described previously (5). Briefly, amplified DNA was hybridized onto a membrane that was covalently pre-coated with a set of 43 spacer oligonucleotides derived from the spacer sequences of *M. tuberculosis* H37Rv and *M. bovis* P3. The ECL Detection system (GE Healthcare, USA) was applied for final image detection. Spoligotypes were compared with the SpolDB4/SITVIT global database (http://www.pasteur-guadeloupe.fr:8081/SITVITDemo/).

### Mycobacterial interspersed repetitive unit variable number tandem repeat (MIRU-VNTR) typing

MIRU-VNTR typing was conducted as previously described (6) with some modifications. The 10-loci MIRU-VNTR included VNTR3820, QUB3232, QUB2163b, Mtub04, VNTR4120, MIRU39, QUB18, QUB-26, Mtub21, and MIRU26. Briefly, the 10 MIRU-VNTR loci were amplified in three multiplex PCR assays, and the sizes of the amplicons were assessed with the GeneScan 1200 LIZ Size Standard (Applied Biosystems, Waltham, MA, USA) by capillary electrophoresis on a sequencer (3500 Genetic Analyze, Applied Biosystems, Waltham, MA, USA). The analysis was carried out using GeneMapper software (Applied Biosystems, Waltham, MA, USA), which assigns alleles based on the customized bin sets (fragment sizes and dyes) used to define each repeat number.

### Whole-genome sequencing

*M. tuberculosis* isolates were subcultured on Middlebrook 7H11 medium (Becton, Dickinson and Company, Spark, MD, USA) at 37°C. Bacterial colonies were resuspended in 1× TE buffer and heat-inactivated at 80°C for 1 hour. Genomic DNA was extracted using the cetyltrimethylammonium bromide (CTAB)-chloroform method, followed by bead clean-up (KAPA HyperPure Beads; Roche Sequencing Solutions, Inc., Pleasanton, CA, USA). Quantitative measurement of the DNA was performed using the Qubit 4 fluorometer (ThermoFisher Scientific, Waltham, MA, USA). Paired-end libraries were prepared using the TruSeq DNA PCR-Free LT Sample Preparation Kit (Illumina, Inc., San Diego, CA, USA) according to the manufacturer’s protocol. The average fragment size (500–600 bp) of the DNA libraries was checked by the Agilent 2100 Bioanalyzer in combination with the High Sensitivity DNA Kit (Agilent Technologies, Inc., Waldbronn, Germany). The concentration of the DNA libraries was measured by quantitative PCR with the KAPA Library Quantification Kit (Roche Sequencing Solutions, Inc., Pleasanton, CA, USA). The 24 purified DNA libraries were pooled, and DNA concentrations were quantified with a Qubit 4 fluorometer. The pooled libraries (11 pM) were sequenced on an Illumina MiSeq system (Illumina, Inc., San Diego, CA, USA) with the MiSeq Reagent Kit ver. 3 (600 cycles).

### Phylogenetic analysis

The overall quality of sequence reads was checked using FastQC (www.bioinformatics.babraham. ac.uk/projects/fastqc/). Verified paired-end reads were trimmed and mapped to the reference genome H37Rv (NC_000962.3) using BioNumerics v7.6 (Applied Maths, Kortrijk, Belgium) with default values. SNP analysis was conducted using BioNumerics v7.6 with strict SNP filtering. The variants filtration was performed based on the following criteria: having total coverage of five reads, not containing ambiguous bases, not containing gaps, and not being within 12 base pairs of adjoining called SNPs. Non-informative SNPs were also excluded from further analysis. SNP positions in the isolates were concatenated to a sequence alignment, excluding SNPs located in repetitive regions of the genome, such as PE/PPE family genes. The median-joining network was conducted by PopART v1.7 software.

## RESULTS

### Characteristics of study clusters

**Fig 2:**
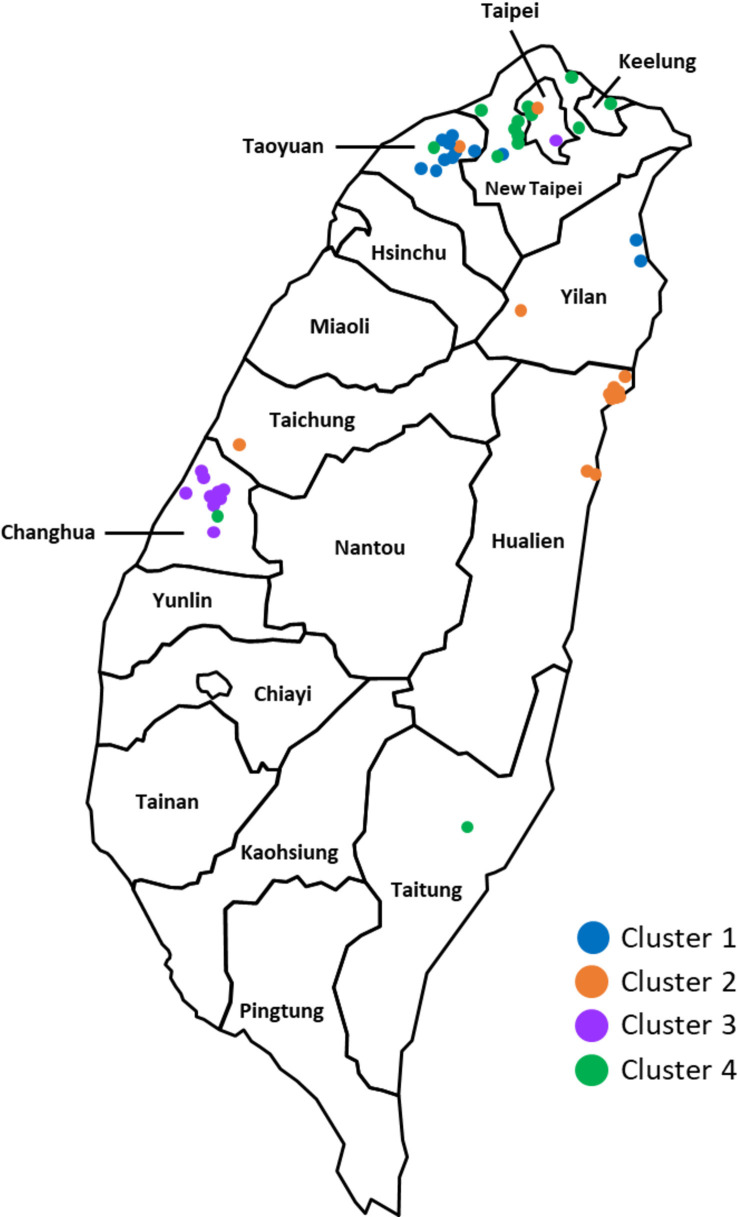
Geographic distribution of studied cases within the four TB clusters in Taiwan. The source of the map is https://blankmapsrepo.com/taiwan-political-map/.

Characteristics of the cases in four selected clusters with thorough epidemiological data are listed in Table 1. Four clusters belonged to the Unknown (C1, C4), Haarlem-3 (C2), and Beijing (C3) genotypes. The majority of cases were male (47.6%–82.1%), new cases (61.9%–96.4%), and pulmonary TB (89.3%–100.0%). The median ages were 19, 36, 68, and 63 years, respectively. The acid-fast bacillus (AFB) smear-positive rates ranged from 21.4% to 66.7%. Geographic distribution of the studied cases is shown in Fig. 2.

**TABLE 1:** Characteristics of 82 study cases in four TB clusters

Characteristics	Cluster 1 (*N* = 28) No. (%)	Cluster 2 (*N* = 21) No. (%)	Cluster 3 (*N* = 12) No. (%)	Cluster 4 (*N* = 21) No. (%)
Collection year	2012–2018	2007–2018	2007–2021	2012–2021
Contact setting	School	Community	Same county	Community, social
Genotype	Unknown	Haarlem-3	Beijing	Unknown
Sex				
Male	23 (82.1)	10 (47.6)	9 (75.0)	16 (76.2)
Female	5 (17.9)	11 (52.4)	3 (25.0)	5 (23.8)
Age				
<25	23 (82.1)	1 (4.8)	1 (8.3)	1 (4.8)
25–44	4 (14.3)	12 (57.1)	0 (0.0)	4 (19.0)
45–64	1 (3.6)	7 (33.3)	3 (25.0)	7 (33.3)
≥65	0 (0.0)	1 (4.8)	8 (66.7)	9 (42.9)
Case category				
New	27 (96.4)	13 (61.9)	8 (66.7)	19 (90.5)
Previously treated	1 (3.6)	8 (38.1)	4 (33.3)	2 (9.5)
Site of TB				
Pulmonary TB	25 (89.3)	21 (100.0)	11 (91.7)	19 (90.5)
Extrapulmonary TB	3 (10.7)	0 (0.0)	1 (8.3)	2 (9.5)
Acid fast bacillus smear				
Positive	6 (21.4)	7 (33.3)	6 (50.0)	14 (66.7)
Negative	22 (78.6)	14 (66.7)	6 (50.0)	7 (33.3)

### WGS-based cluster investigation

#### An aggregate-setting TB outbreak

**Fig 3:**
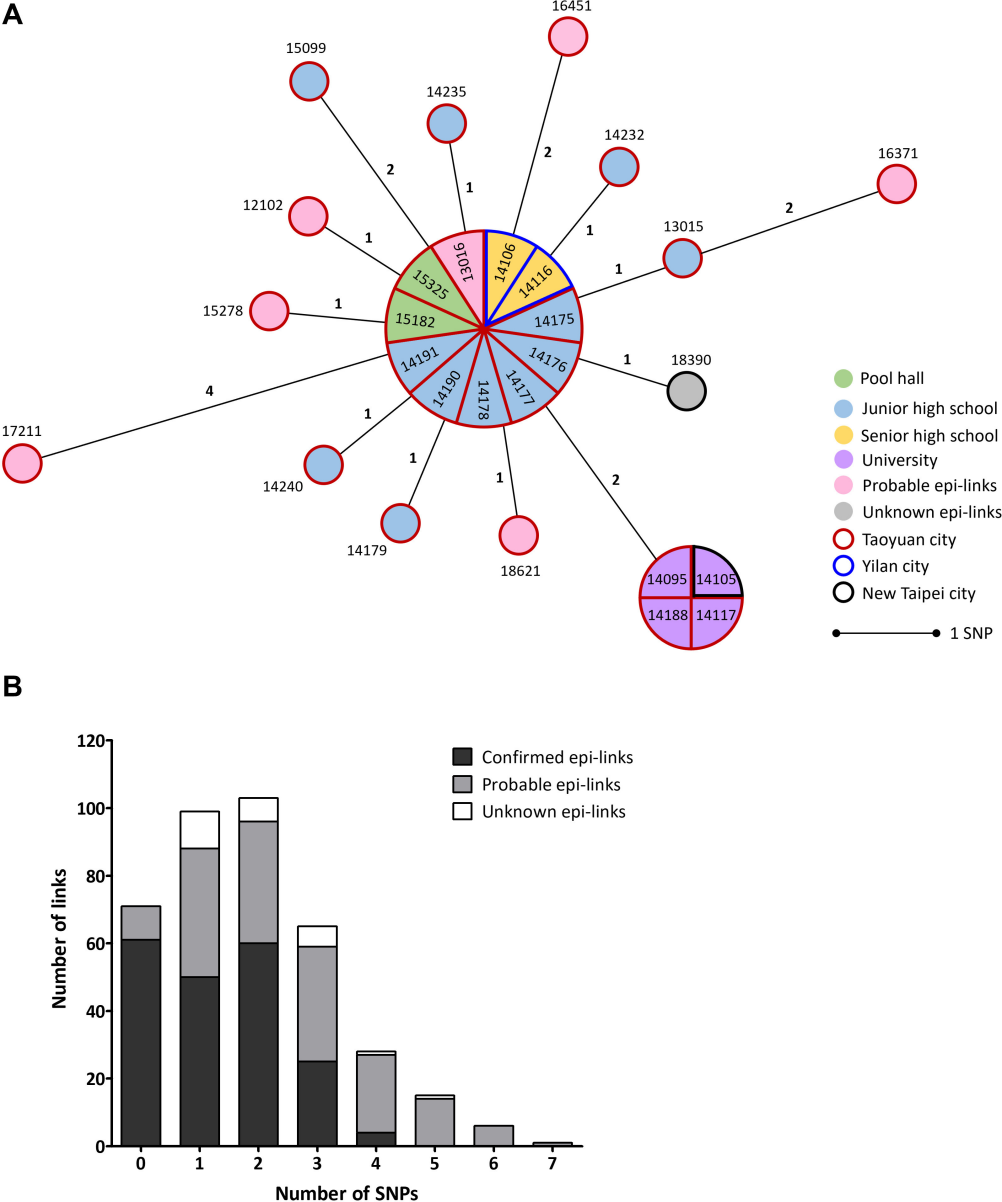
(**A**) Median-joining network of Cluster 1 consisted of 28 isolates. The circles represent *M. tuberculosis* isolates (numbered according to case identification). Isolates are separated by the lines with length representing SNP distance. Isolates with identical genomes are grouped in the same circle. Epidemiological links between cases are labeled with different colors within each node, while geographical regions where cases lived are labeled with colors outside each node. (**B**) Distribution of the number of SNPs in epidemiological links between cases of Cluster 1.

Cluster 1 (C1) was an Unknown spoligotype TB cluster consisting of 28 cases (Fig. 3A). Cases infected with strains with 0 SNP difference were found in settings including a pool hall (*N* = 2), a junior high school in Taoyuan city (*N* = 6), a senior high school in Yilan city (*N* = 2), and a university in Taoyuan city (*N* = 4). Since cases were from separate settings, cases seemed to be unrelated initially. Nevertheless, combining the results of contact investigations, the outbreak was confirmed. Cases 15182, 14106, and 14095 all attended the same junior high school in Taoyuan city with 0–4 SNPs difference. Six cases from proximate geographic locations with probable epi-links differed by 1–7 SNPs. This might be due to spillover transmission to the community. Nevertheless, case 18390 from a different city with an unknown epi-link was excluded.

All the epidemiologically confirmed isolates fell within a 5-SNP threshold (Fig. 3B). Therefore, in an aggregate setting, the 5-SNP threshold could be applied to confirm an outbreak for inferring a definite epi-link. In addition, the isolate with probable epi-links had an SNP difference of 7, implying more widespread transmission.

#### An MDR-TB cluster originated in a high TB prevalence area

**Fig 4:**
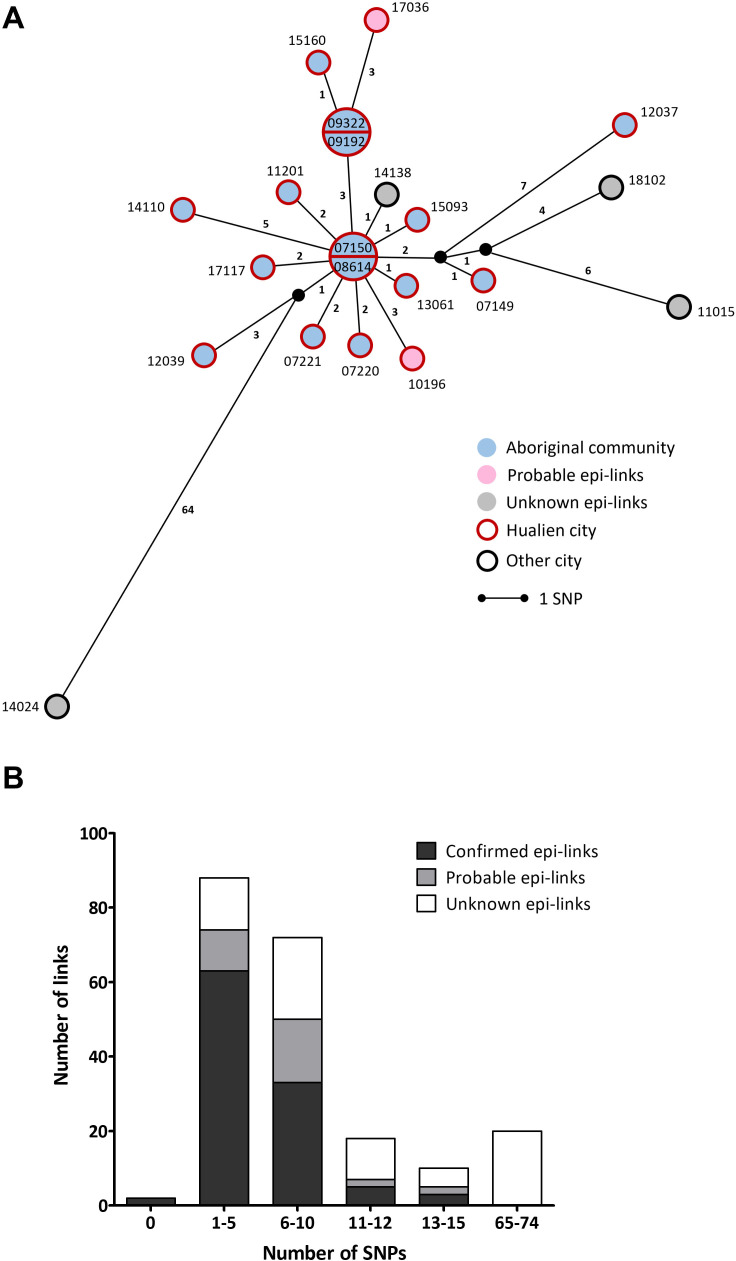
(**A**) Median-joining network of Cluster 2 consisted of 21 isolates. The circles represent *M. tuberculosis* isolates (numbered according to case identification). Isolates are separated by the lines with length representing SNP distance. Isolates with identical genomes are grouped in the same circle. Epidemiological links between cases are labeled with different colors within each node, while geographical regions where cases lived are labeled with colors outside each node. (**B**) Distribution of the number of SNPs in epidemiological links between cases of Cluster 2.

Cluster 2 (C2) was a Haarlem-3 spoligotype MDR-TB cluster identified in an aboriginal community located in Eastern Taiwan over a time span of 11 years (2007–2018). C2 comprised 21 TB cases, 15 of which were from the same aboriginal community and their isolates had 0–14 SNPs difference (Fig. 4A). The villagers encountered frequently and had close contacts. A total of 10 known epi-links between villagers were identified through routine contact tracing (one workplace contact, seven household contacts, and two social contacts), which differed by 1–13 SNPs (Table S1). In addition, cases 10196 and 17036 were from the same city but not from the aboriginal community mentioned above. These two cases had 3–15 SNPs from those of 15 aboriginal villagers, suggesting that transmission may have spread from the community. Nevertheless, the remaining four cases from four different cities with unknown epi-links were excluded.

In C2, cases with confirmed or probable epi-links had ≤15 SNPs difference between isolates (Fig. 4B). Using the 12-SNP threshold, case 12037 was excluded based on 13–14 SNPs difference from three cases (12039, 15160, and 14110). However, epidemiological investigations showed that these four cases were from the same aboriginal community with close relationships. Since none of the epi-linked cases had isolates separated by more than 15 SNPs. Therefore, we suggested using 15 SNPs as a threshold, especially when defining a long-term and/or an MDR-TB outbreak.

#### An MDR-TB cluster with spatial and geographical relationship

**Fig 5:**
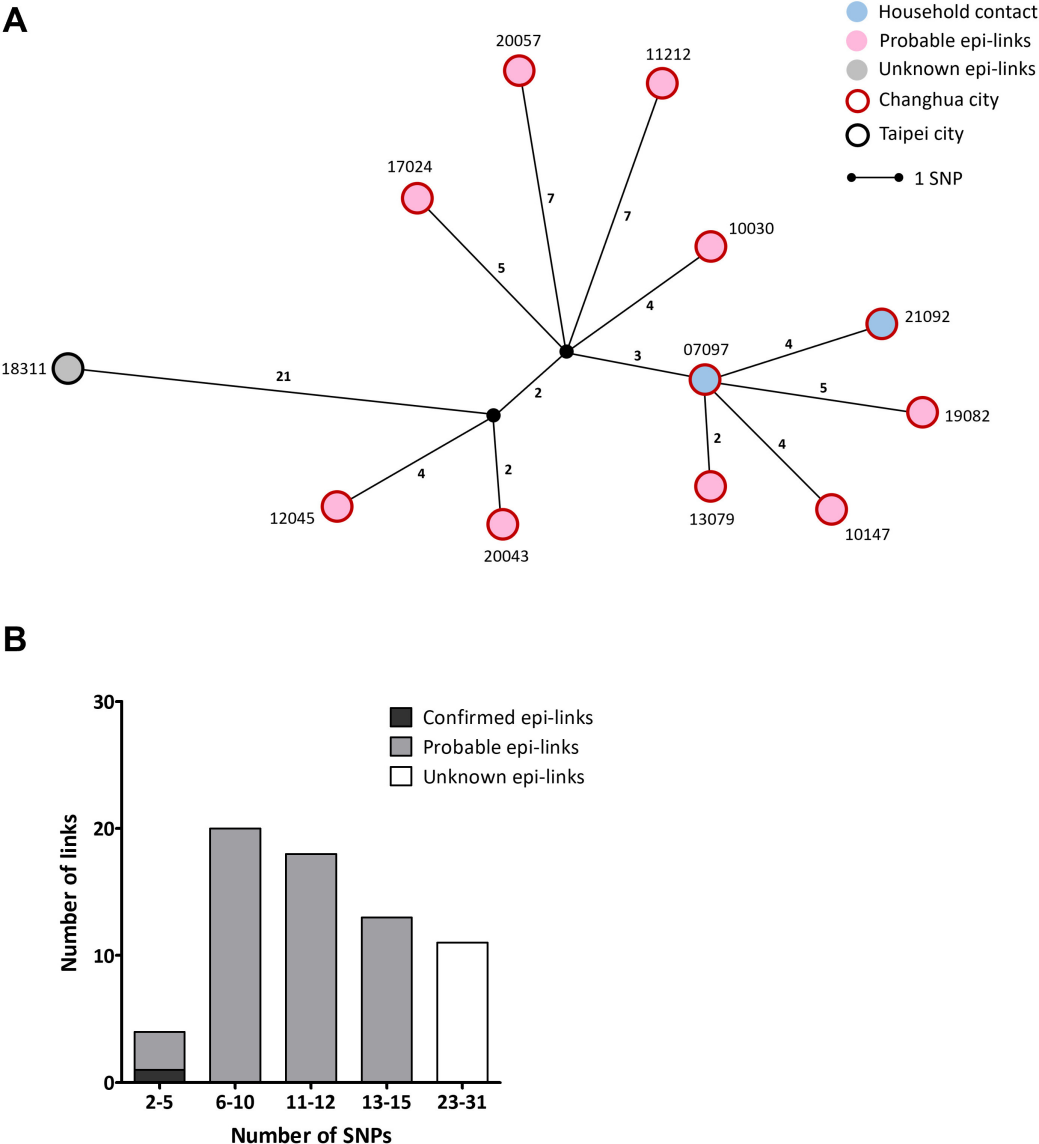
(**A**) Median-joining network of Cluster 3 consisted of 12 isolates. The circles represent *M. tuberculosis* isolates (numbered according to case identification). Isolates are separated by the lines with length representing SNP distance. Epidemiological links between cases are labeled with different colors within each node, while geographical regions where cases lived are labeled with colors outside each node. (**B**) Distribution of the number of SNPs in epidemiological links between cases of Cluster 3.

Cluster 3 (C3) was a Beijing spoligotype MDR-TB cluster that comprised 12 cases, 11 of which were from the same city. These cases lived in neighboring townships and their isolates had ≤15 SNPs difference (Fig. 5A). Cases 07097 and 21092 were the same household contacts with four SNPs difference. One remaining case from a different city with an unknown epi-link differed by 23–31 SNPs. In C3, cases with confirmed or probable epi-links had ≤15 SNPs difference between isolates (Fig. 5B). Using the 12-SNP threshold would exclude the geographically related cases. None of the epi-linked cases had isolates separated by more than 15 SNPs. Considering that the C3 cluster is an MDR-TB cluster, and the onset period was 15 years, we suggested using a threshold with 15 SNPs difference for inferring a probable outbreak.

#### An MDR-TB cluster originated in a community

**Fig 6:**
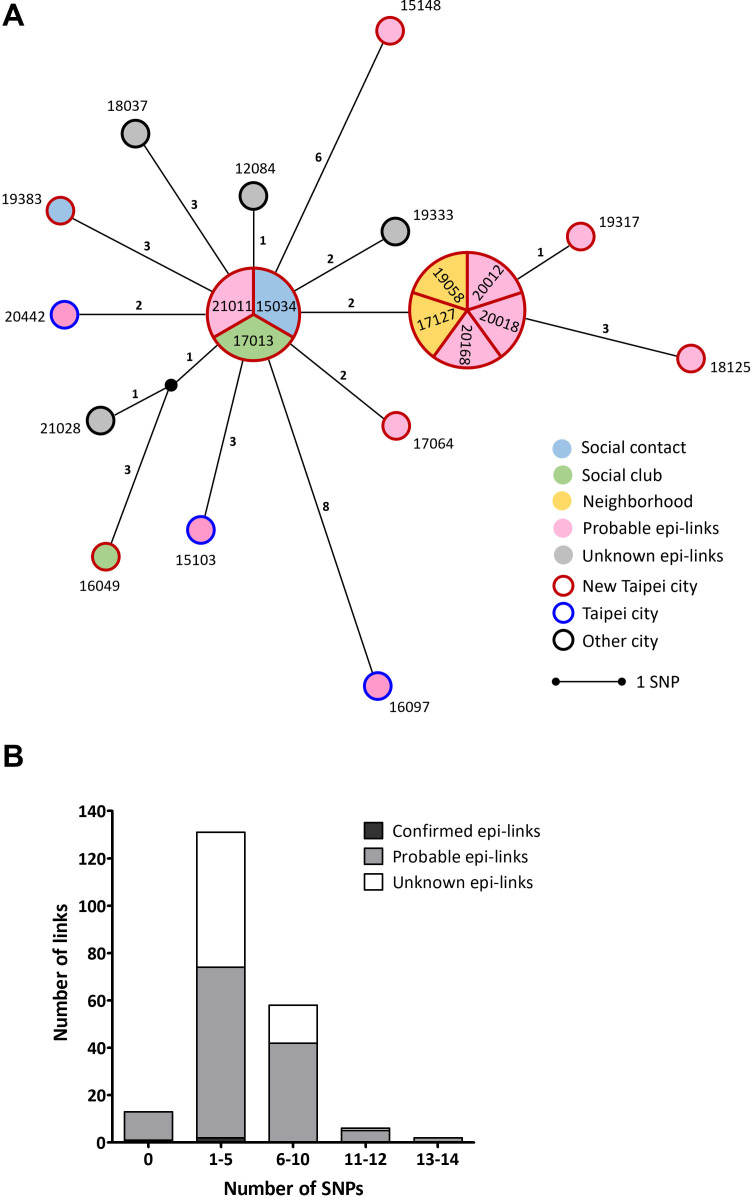
(**A**) Median-joining network of Cluster 4 consisted of 21 isolates. The circles represent *M. tuberculosis* isolates (numbered according to case identification). Isolates are separated by the lines with length representing SNP distance. Isolates with identical genomes are grouped in the same circle. Epidemiological links between cases are labeled with different colors within each node, while geographical regions where cases lived are labeled with colors outside each node. (**B**) Distribution of the number of SNPs in epidemiological links between cases of Cluster 4.

Cluster 4 (C4) was identified as an Unknown spoligotype MDR-TB cluster which comprised 22 cases. Five cases (15034, 17013, 20012, 19383, and 16049) lived in the same community, and two known epi-links were identified through social contacts with 3–4 SNPs differences (Fig. 6A). Although 17127 was from another district, he has been previously lived in the same community for 30 years, suggesting that there may be an epi-link between 17127 and the other cases in C4. Consequently, 17127 caused spillover transmission to a neighbor, case 19058, whose isolate was identified by zero SNP difference with 17127. Cases with probable epi-links had 0–14 SNPs difference. Nevertheless, the remaining four cases from four different cities with unknown epi-links were excluded.

In C4, cases with confirmed or probable epi-links had <15 SNPs difference between isolates (Fig. 6B). Using the 12-SNP threshold would exclude three geographically related cases (15148, 16097, and 18125). We found that the 15-SNP threshold could be used to identify cases in a putative outbreak with geographical relationships. Therefore, the appropriate threshold is 15 SNPs difference for inferring a probable epi-link.

### SNP thresholds for delineation of transmission

**Fig 7:**
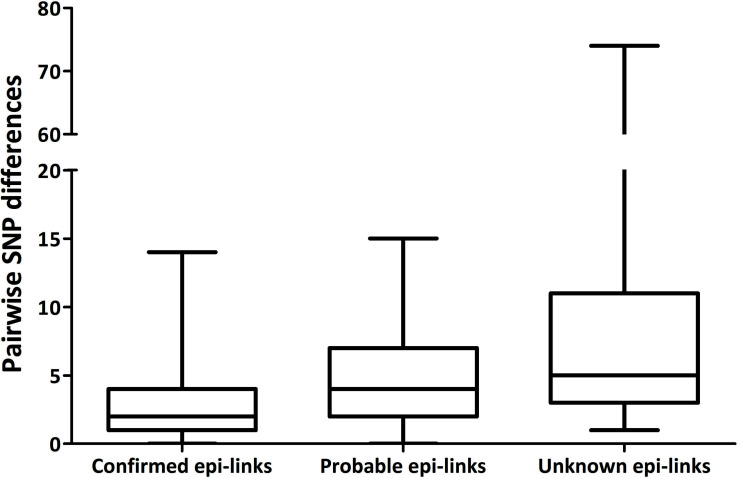
Box plot of pairwise SNP differences between isolates with different strengths of epi-links.

Table 2 shows the SNP thresholds used in the previous studies to define a cluster. Walker et al. (11) investigated SNP differences within community and household clusters in the UK, concluding that epi-link consistent with transmission existed between isolates with ≤5 SNPs difference, and not existed between isolates with >12 SNPs difference. Studies have employed these pre-defined SNP thresholds to define transmission clusters (12–14). An alternative approach determined the variation between improbable transmission pairs first and, as no pair had <2 SNPs difference, used 0–1 SNPs between isolates to define a cluster (15). Xu et al. (16) used a cutoff of ≤15 SNPs to define recent and previous transmission events. In this study, pairwise SNP distances and strength of epi-link were plotted (Fig. 7). All pairs with confirmed epi-links had <15 pairwise SNPs, and 75.0% of these pairs had <5 pairwise SNPs. In addition, all pairs with probable epi-links had ≤15 pairwise SNPs. We determined thresholds of ≤5 and ≤15 SNPs difference to categorize either a definite or a probable TB cluster surveillance for further outbreak investigations.

**TABLE 2:** Studies using SNP thresholds to delineate transmission

Authors	Lower SNP threshold	Upper SNP threshold
Clark et al. (17)	<50	>50
Guerra-Assunção et al. (18)	≤10	(Not specified)
Lee et al. (15)	<2	(Not specified)
Roetzer et al. (8)	≤3	(Not specified)
Walker et al. (11)	≤5	>12
Yang et al. (19)	≤10	(Not specified)
Xu et al. (16)	≤15	(Not specified)
This study	≤5	≤15

## DISCUSSION

WGS-based genotyping offers valuable opportunities for enhancing the detection and tracking of outbreaks and transmission events, leading to the precise execution of public health actions (20). The establishment of an optimal SNP threshold is crucial for accurately delineating TB transmission. Taiwan remains a medium TB burden county with an incidence rate of 28 per 100,000 population in 2022 (2). For Ending TB, understanding and halting TB transmission is needed to transform evidence to stringent practices. In our study, four diverse and representative clusters, including a high-risk population, a conjugated setting, an MDR-TB group, and a community, pre-defined using less-discriminatory spoligotyping and MIRU-VNTR typing methods. We found that 75.0% of paired isolates with confirmed epi-links had <5 pairwise SNPs, while all paired isolates with probable epi-links had ≤15 pairwise SNPs. Consequently, we concluded that thresholds of ≤5 and ≤15 SNPs difference between isolates were used to categorize definite and probable TB transmission, respectively.

SNP-based clustering has been applied to track transmission chains with fidelity. However, it is worth noting that SNP thresholds for *M. tuberculosis*, as described in the literature, could vary widely (9). The thresholds of 5 or 12 SNPs were frequently used to suggest epi-links, particularly in studies from regions with low TB incidence and low drug resistance (9). In the UK, where non-UK-born patients displayed a high level of clustering without identified epi-links, WGS was employed to delineate transmission networks by combining information on SNPs as a measure of epidemiological data (21). Similarly, in Europe, cross-border clusters with an increased likelihood of recent transmission were identified. The WGS-based surveillance system could efficiently elucidate the dynamics of in-country and cross-border TB transmission across European countries (22). In Australia, TB clusters were predominantly attributed to local transmission, with most instances occurring beyond household contacts. Routine WGS, using a 5-SNP threshold, facilitated the monitoring of local TB transmission and guided targeted public health interventions (23, 24). Understanding the transmission of MDR *M. tuberculosis* strains is crucial for eliminating TB in high-incidence countries. Studies conducted in China suggest combining genomic, epidemiological, and spatial analysis to identify transmission hotspots and investigate transmission dynamics (25, 26). Southern Africa with a high TB burden also integrated genomic and geospatial data to identify geographically clustered *M. tuberculosis* strains, representing localized areas of recent transmission (27, 28). Previous studies demonstrated that cases with confirmed epi-links generally exhibit ≤5 SNPs difference, leading to the proposal of a 5-SNP threshold to define highly related isolates (29), as supported by our study findings. A 12-SNP threshold has been used to indicate potentially related isolates (29). Nevertheless, our results found that even a 12-SNP threshold may miss certain cases linked by epidemiological or geographical relationships, highlighting the complexity of transmission in different settings. In our study, there were three long-term transmission clusters of MDR-TB, prompting the suggestion of a maximum threshold of 15 SNPs for defining genomic relatedness between cases. A population-based study conducted in the Valencia Region of Spain also employed a 15-SNP threshold to include recent and old transmission events (16). Cancino-Muñoz et al. suggested that a 12-SNP threshold may not be valid in every setting when analyzing transmission dynamics, particularly in regions with a higher transmission burden (30).

The appropriate SNP thresholds could be affected by multiple factors (29, 31). The *M. tuberculosis* mutation rates have been estimated at 0.5 SNPs per genome per year, and this rate could vary significantly, especially in regions with high TB and DR-TB prevalence (32). Antibiotic resistance and selective pressure could increase the mutation rate of more than three SNPs (32). This increase may be attributed to specific genomic loci, both in genes associated with drug resistance and compensatory mutations, exhibiting a mutation rate higher than the background (33). Moreover, the phylogeographic lineage of strains could potentially impact the mutation rate, with lineage 2 having a higher mutation rate (34). Within-host microevolution further complicated the SNP distance between isolates, posing challenges in the interpretation of transmission events (35). In a study on a large isoniazid-resistant TB outbreak in London, WGS analysis of 27 individual colonies cultured from a single patient specimen showed a maximum genetic distance of 6 SNPs between any pair (36). At one extreme, up to 50 SNP differences have been reported to occur over throughout the infection in patients with advanced disease when sampling from multiple body sites (37).

Genomic regions containing homopolymers or tandem repeats may lead to false reports of indels or SNPs due to sequencing errors or difficulties in read mapping (38). Current pipelines for inferring transmission typically excluded indels or variants detected in repetitive or duplicated regions (38). However, with the advent of more accurate variant callers, parameters, and long-read sequencing, the inclusion of such sites provides further resolution for outbreaks and potentially alters current SNP thresholds for defining transmission clusters in the future (10). Furthermore, previous results revealed significant differences in sequencing methods, raw read data processing, quality control criteria for SNP calling, and phylogenetic algorithms across different studies (39). These diversities in pipelines could impact the number of SNP differences between isolates and pose challenges in comparing WGS results between different laboratories and pipelines (39).

The definition of SNP thresholds for inferring transmission relies on the availability of detailed epidemiological data. One limitation of our study is the presence of missing epi-links among certain cases within the same cluster. This scarcity of epidemiological data can be attributed to variations in the onset times of cases, spatial migrations, and the inherent challenge of identifying all potential contacts. Infected contacts may exhibit active infection soon after contact, may not reactivate until much later in life, or may never become ill with TB, making transmission chains more complicated to interpret. Another limitation is the lack of spatial analysis of transmission, impeding a comprehensive understanding of the transmission dynamics. In the future, we plan to employ Geographic Information Systems (GIS) to conduct spatial analysis to enhance molecular epidemiological surveillance. The other limitation is that our study is not population based, approximately 20% are culture-negative TB cases and 50% of culture-positive TB cases were genotyped, potentially leading to underestimating the SNP thresholds.

Our results illustrate that the WGS-based approach helps rule in and rule out transmission with greater discriminatory power than MIRU-VNTR, thereby facilitating outbreak investigations. However, considering cost-effectiveness, MIRU-VNTR can still serve as the primary genotyping method to rule out transmission. In cases where isolates have identical MIRU-VNTR genotypes, WGS can be employed as the secondary genotyping method to either confirm or refute transmission. While phylogenetic trees portray useful information about sequence relatedness, they alone may not be sufficient to depict the network of actual transmission events. Therefore, the inclusion of epidemiological and demographic data remains critical to outbreak investigations.

Conventional genotyping methods overestimate TB transmission, hampering precise detection of TB outbreaks and comprehensive surveillance. To strengthen and revolutionize the TB elimination program, WGS offers significant benefits for delineating transmission networks, conducting molecular surveillance, and advancing genomic epidemiology. This study demonstrated the use of thresholds of ≤5 and ≤15 SNP differences between isolates to categorize definite and presumable TB transmission, respectively. Different SNP thresholds should be applied while a higher cutoff was required to define an MDR-TB outbreak. The utilization of SNP thresholds proves to be a valuable tool for guiding public health interventions, eliminating the need for unnecessary public health actions, and potentially uncovering undisclosed TB transmissions. Our results can serve as a guide to enhance outbreak investigations, ultimately striving zero TB transmission.

## Data Availability

Sequencing reads have been submitted to the National Center for Biotechnology Information (NCBI) Sequence Read Archive (SRA) under BioProject ID PRJNA1065624.

## SUPPLEMENTAL MATERIAL

Supplemental material spectrum.00418-24-s0001.docx Table S1.

ASM does not own the copyrights to Supplemental Material that may be linked to, or accessed through, an article. The authors have granted ASM a non-exclusive, world-wide license to publish the Supplemental Material files. Please contact the corresponding author directly for reuse.
