# Supplementary material for: Define SNP thresholds for delineation of tuberculosis transmissions using whole-genome sequencing
Source: Microbiol Spectr. 2024 Jun 25;12(8):e00418-24. doi: 10.1128/spectrum.00418-24 (PMC11302064; doi:10.1128/spectrum.00418-24)
Supplement: Supplemental material — Table S1. [file spectrum.00418-24-s0001.docx]

**Supplementary Table**

| **TABLE S1** Epidemiological links identified in cases of Cluster 2 | | |
| --- | --- | --- |
| **Link** | **SNP difference** | **Contact setting** |
| 07150-07149 | 3 | Workplace |
| 07150-07220 | 2 | Household |
| 07221-13061 | 3 | Household |
| 07221-14110 | 7 | Household |
| 13061-14110 | 6 | Household |
| 09322-12039 | 7 | Household |
| 09322-15160 | 1 | Household |
| 12039-15160 | 8 | Household |
| 11201-17117 | 4 | Social |
| 12037-12039 | 13 | Social |
